# A Whole Brain Staining, Embedding, and Clearing Pipeline for Adult Zebrafish to Visualize Cell Proliferation and Morphology in 3-Dimensions

**DOI:** 10.3389/fnins.2017.00750

**Published:** 2018-01-17

**Authors:** Benjamin W. Lindsey, Alon M. Douek, Felix Loosli, Jan Kaslin

**Affiliations:** ^1^Australian Regenerative Medicine Institute, Monash University, Clayton, VIC, Australia; ^2^Institute of Toxicology and Genetics, Karlsruhe Institute of Technology, Karlsruhe, Germany

**Keywords:** macro-imaging, tissue clearing, medaka, optical projection tomography, neurogenesis, stem cell senescence, regeneration, development

## Abstract

The field of macro-imaging has grown considerably with the appearance of innovative clearing methods and confocal microscopes with lasers capable of penetrating increasing tissue depths. The ability to visualize and model the growth of whole organs as they develop from birth, or with manipulation, disease or injury, provides new ways of thinking about development, tissue-wide signaling, and cell-to-cell interactions. The zebrafish (*Danio rerio*) has ascended from a predominantly developmental model to a leading adult model of tissue regeneration. The unmatched neurogenic and regenerative capacity of the mature central nervous system, in particular, has received much attention, however tools to interrogate the adult brain are sparse. At present there exists no straightforward methods of visualizing changes in the whole adult brain in 3-dimensions (3-D) to examine systemic patterns of cell proliferation or cell populations of interest under physiological, injury, or diseased conditions. The method presented here is the first of its kind to offer an efficient step-by-step pipeline from intraperitoneal injections of the proliferative marker, 5-ethynyl-2′-deoxyuridine (EdU), to whole brain labeling, to a final embedded and cleared brain sample suitable for 3-D imaging using optical projection tomography (OPT). Moreover, this method allows potential for imaging GFP-reporter lines and cell-specific antibodies in the presence or absence of EdU. The small size of the adult zebrafish brain, the highly consistent degree of EdU labeling, and the use of basic clearing agents, benzyl benzoate, and benzyl alcohol, makes this method highly tractable for most laboratories interested in understanding the vertebrate central nervous system in health and disease. Post-processing of OPT-imaged adult zebrafish brains injected with EdU illustrate that proliferative patterns in EdU can readily be observed and analyzed using IMARIS and/or FIJI/IMAGEJ software. This protocol will be a valuable tool to unlock new ways of understanding systemic patterns in cell proliferation in the healthy and injured brain, brain-wide cellular interactions, stem cell niche development, and changes in brain morphology.

## Introduction

How cells in the developing or adult brain are organized and behave following injury or disease remains a fascinating, yet still poorly understood, question. For many years, the ability to visualize the structural composition or global patterns across the neuro-axis of the adult vertebrate brain was limited by the lack of imaging tools available to examine cell phenotypes in larger tissue structures *in situ* in 3-dimensions (3-D). As a result, most interpretations have been derived from 2-dimensional (2-D) analyses of sectioned tissue. Recently however, the field of macro-imaging has become popularized by a growing interest of researchers to understand whole organ development, structure, and the associated morphological and cellular abnormalities that arise with disease (Short et al., 2010; Epp et al., 2015; Lloyd-Lewis et al., 2016; Short and Smyth, 2016). This has been paralleled by innovations in modern clearing techniques and specialized imaging methods designed to visualize thick tissues or whole organs in 3-D space, giving way to a new era of fluorescent, whole organ imaging (Susaki et al., 2014; Azaripour et al., 2016; Susaki and Ueda, 2016; Aswendt et al., 2017; Whitehead et al., 2017).

The value of macro-imaging has been demonstrated across a range of tissues, including embryos (Sharpe et al., 2002; Sharpe, 2003), heart (Kolesová et al., 2016; Aguilar-Sanchez et al., 2017), kidney (Short et al., 2010; Combes et al., 2014; Short and Smyth, 2016, 2017), lymph node (Song et al., 2015), mammary glands (Lloyd-Lewis et al., 2016), and brain (Gleave et al., 2013; Ode and Ueda, 2015), leading to new insight into the cellular behavior of organs under diverse conditions. This progress has been facilitated by the power of multiphoton imaging, newer confocal microscopes with lasers having increasingly better z-axis penetration, the development of light-sheet microscopes, and tomographic techniques such as Optical Projection Tomography (Sharpe et al., 2002; Keller et al., 2010; Parra et al., 2012; Kromm et al., 2016; McGowan and Bidwell, 2016; Susaki and Ueda, 2016; Whitehead et al., 2017). Nevertheless, whole organ imaging of thick tissue of ~1 mm or greater introduce a number of challenges that must be overcome compared to antibody labeling and confocal imaging of sectioned tissue at the micron scale.

In most cases the biggest obstacle for macro-imaging is the successful sample preparation of thick tissue or organs. A significant challenge continues to be the balance between homogeneous fluorescent labeling through the tissue block and rendering the tissue clear for imaging. Unfortunately, this can only be accomplished by trial and error, with individual tissue types having their own unique set of physical properties. Commonly protein labeling using antibodies or transgenic reporter lines, such as Green Fluorescent Protein (GFP), show excellent fluorescent signal prior to clearing steps. However, reagents used for transitioning tissue to a cleared state often reduce fluorescence levels or quench fluorescence altogether. To circumvent this problem, a variety of different tissue clearing methods have been developed, making use of CLARITY-based methods (i.e., PARS, PACT; Chung and Deisseroth, 2013; Yang et al., 2014; reviewed in Vigouroux et al., 2017), aqueous methods (i.e., CUBIC, Scale; Susaki et al., 2015), and non-aqueous methods such as 3DISCO (Belle et al., 2014, 2017; reviewed in Vigouroux et al., 2017), iDISCO (Renier et al., 2014), uDISCO (Pan et al., 2016), and BABB (Ahnfelt-Rønne et al., 2007). While the success of these methods appear to vary by tissue, some indeed show promise for preserving fluorescence for downstream imaging.

Many of the above clearing methods have been established specifically for studies of neural-circuitry or cell-specific analysis in the mammalian brain (Parra et al., 2012; Chung and Deisseroth, 2013; Susaki et al., 2014; Epp et al., 2015; reviewed in Azaripour et al., 2016; Vigouroux et al., 2017). However, the large size of the adult brain of rodent models, can limit imaging options or restrict imaging of the brain to only a specific subregion during a single scan. Unlike the rodent brain, the smaller brain of teleost fishes such as zebrafish and medaka, show exceptional promise as experimental models to visualize spatial changes along the 3-D neuro-axis in adulthood under physiological or compromised states. Having the opportunity to investigate cell dynamics within a 3-D context offers the chance to address novel questions concerning cell-specific behavior, systemic signaling, stem cell niche development, and morphological variation.

The zebrafish, in particular, has become a rising star in the field of adult neurogenesis, plasticity, and regeneration (Kaslin et al., 2008; Kizil et al., 2012; Lindsey and Tropepe, 2014; Lindsey et al., 2014; Than-Trong and Bally-Cuif, 2015; Alunni and Bally-Cuif, 2016; Ghosh and Hui, 2016). Constitutively cycling adult neural stem cells are found across an extensive number of neurogenic compartments along the anterior-posterior (A-P) neuro-axis (Adolf et al., 2006; Grandel et al., 2006; Chapouton et al., 2007), with these cells capable of producing newly regenerated neurons following brain injury (Kroehne et al., 2011; Baumgart et al., 2012; Kishimoto et al., 2012; Kyritsis et al., 2012; Kaslin et al., 2017). With its well mapped neurogenic niches, heterogeneous mixture of adult stem cell populations (Ganz et al., 2010; Lindsey et al., 2012), array of cell-specific transgenic lines, molecular toolbox, and highly conserved genome compared with its vertebrate counterparts, the zebrafish provides an exquisite experimental system to uncover clues governing stem cell dynamics under homeostasis and regeneration. However, there has yet to be developed a straightforward method to label and visualize adult stem cell populations in a 3-D context. While highly transparent zebrafish mutants lacking skin pigmentation, such as *casper* and *crystal*, have been made available in recent years, these lines only benefit imaging of larvae or early juvenile stages (White et al., 2008; Antinucci and Hindges, 2016), but do not mitigate opacity and light scattering in the adult brain. As a result, developing new clearing and imaging methods permitting 3-D visualization of changes in cell proliferation tailored for the adult zebrafish brain are needed. Therefore, the rationale for establishing the protocol described herein was to develop an efficient and feasible method to visualize and analyze actively cycling adult stem cells in their respective niches, in order to understand how they respond at a global scale to brain injury or when shifted from a homeostatic state.

Our protocol describes an 8-step method for sample preparation in advance of Optical Projection Tomography (OPT) imaging. The protocol takes advantage of the small molecular size of 5-ethynyl-2′-deoxyuridine (EdU) to reliably label cells in the *S*-phase of the cell cycle and uses individual reagents to avoid the high cost of the commercial “Click-it EdU kit” for staining (Salic and Mitchison, 2008). Clearing of the entire adult zebrafish brain without loss of EdU fluorescent signal is completed using a combination of benzyl benzoate and benzyl alcohol (BABB, also known as “Murray's reagent”), inexpensive, non-aqueous reagents that have been successfully demonstrated to render tissue transparent (Miller et al., 2005; Zucker, 2006; Short et al., 2010; Gleave et al., 2012, 2013). Unlike many lengthy labeling and clearing protocols for thick tissue, our pipeline allows for the proliferative pattern of samples to be readily visualized and analyzed in less than 10 days using software such as IMARIS or FIJI/IMAGEJ. Moreover, following EdU staining, we highlight that the option of whole brain fluorescent labeling of proteins or transgenic reporter lines can be performed, broadening the applications of this protocol. Finally, we present three analysis methods that can be applied to reconstructed OPT-scanned brain samples that can be completed using FIJI/IMAGEJ. OPT allows fluorescent or non-fluorescent imaging of paraformaldehyde fixed specimens with thicknesses of up to ~15 mm at near cellular resolution (3.21 um/pixel; Sharpe et al., 2002). Three or more fluorescent channels can be sequentially scanned from ultraviolet to infrared (i.e., 350, 488, 555, 647), in addition to brightfield, that can be reconstructed to obtain a 3-D or 2-D view of brains for manipulation. OPT has the added advantage over many newer microscopy techniques in obtaining isotropic datasets that are designed for 3-D volumetric analysis and morphometrics. Taken together, this protocol is the first of its kind to offer a streamlined method of whole brain imaging in the adult zebrafish brain, with the potential to be applied to other small teleost models (i.e., medaka and killifish) or for imaging using modern light-sheet microscopy.

## Materials and methods

All animal experiments were assessed and approved by the Monash University Animal Ethics Committee and were conducted under applicable Australian laws governing the care and use of animals for scientific research. Zebrafish (*Danio rerio*) were maintained in line with standard protocols at the Monash University FishCore.

Medaka (*Oryzias latipes*) stocks were maintained at the Institute of Toxicology and Genetics (ITG) of the Karlsruhe Institute of Technology (KIT). Animal husbandry and experimental procedures were performed in accordance with local and European Union animal welfare standards (Tierschutzgesetz 111, Abs. 1, Nr. 1, AZ35-9185.64/BH). The facility is under the supervision of the local representative of the animal welfare agency.

**Figure 1:**
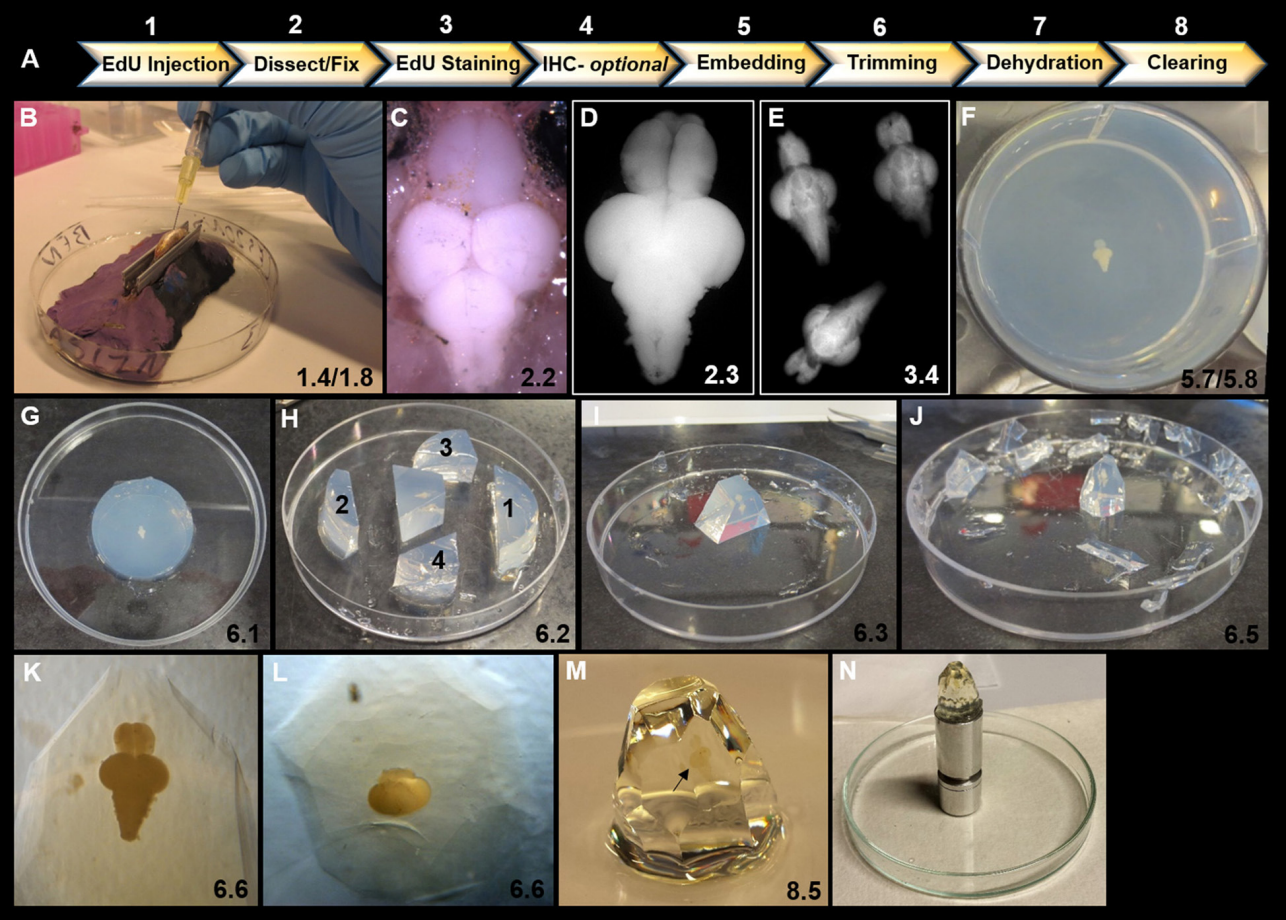
Overview of key steps in sample preparation for optical projection tomography (OPT). **(A)** Summary of 8 step workflow for sample preparation for OPT scanning. **(B)** Intraperitoneal injection of 40 μL of EdU into ventral abdomen of adult zebrafish using a 1 mL syringe and 30 gauge × ½ inch needle. Note the use of V-shaped holder to orient and stabilize the anesthetized specimen during injection. **(C)** Dorsal view of adult zebrafish brain *in situ* prior to excision and fixation. **(D)** Excised adult zebrafish brain fixed in 2% paraformaldyhyde. **(E)** Representative image of three adult brains in EdU staining solution in a 12-well plate. **(F)** Adult brain embedded dorsally and centered in well and in z-plane in low melting agarose in a 6-well plate. **(G)** Low melting agarose cylinder removed from 6-well plate in preparation for trimming. **(H)** Initial trimming using a razor blade to form a trapezoid by 4 sequential cuts: (1) perpendicular to olfactory bulbs, (2) perpendicular to and ~1 cm from spinal cord, and (3 and 4) two lateral diagonal cuts joining 1 and 2 together. **(I)** Trapezoid oriented upright with brain positioned along the long-axis vertically. Olfactory bulbs are localized at the top of the block. **(J)** Trimmed block ready for dehydration and clearing. Notice block is tapered from top to bottom to reduce agarose around brain sample for scanning and to provide a larger base to adhere to mount. **(K–L)** Position of brain within trimmed block viewed under brightfield observed along the long-axis **(K)** and from the dorsal aspect of the block **(L)**. **(M)** Adult zebrafish brain (black arrow) observed *en block* following methanol dehydration and BABB clearing. Note the transparent nature of the brain. **(N)** Sample adhered to an OPT mount in preparation for scanning. In all panels, the corresponding detailed protocol steps are denoted in the bottom right-hand corner.

A summary of the workflow for parts 1–8 is shown in Figure 1A along with representative images of specific steps in the protocol. To facilitate laboratories in adopting this protocol, we have additionally designed a video demonstrating these key steps and procedures (Supplementary Video 1).

### PART 1: labeling proliferating cells using EdU

1.1 Prepare a 10 mM stock of 5-ethynyl-2′-deoxyuridine (EdU) by combining 50 mg of EdU powder (ThermoFisher; A10044) and 20 mL of 1X-Phosphase Buffered Saline (1X-PBS, pH 7.4). Use low heat and vortex as required to dissolve powder in solution.
1.2 Prepare 40 μL aliquots in 0.5 mL PCR tubes (Axygen; 70001981) for injection. This volume of EdU is ideal for intraperitoneal injections of animals between 6-months and 1-year. **Note**: If using newly thawed aliquots of EdU, be sure that the EdU powder has not separated out of solution. If so, use low heat and vortex to reconstitute. Always store EdU solution at −20°C away from light until use. Thawed EdU aliquots should be used within 1–2 days.
1.3 Prepare a solution of 0.04% Tricaine (Sigma; E10521): fish water (i.e., aquarium water) to anesthetize fish prior to injection and setup a separate tank of clean fish water to transfer fish post-EdU injection. Be sure to label tanks clearly.
1.4 Setup a Petri dish for intraperitoneal injections under a dissecting microscope with a large working distance or at the bench. **Note:** To facilitate injections, use a Petri dish with plasticine/modeling clay molded to form a trough lined by the blunt ends of two razor blades (Figure 1B). This V-shaped holder allows adult zebrafish to be quickly and easily placed ventral side up following anaesthetization for EdU injection, without the need to use dissecting tools to orient and hold the fish.
1.5 Draw up a single 40 μL aliquot of EdU solution using a 30 gauge × ½ inch needle (Terumo) into a 1 mL syringe and ensure no air bubbles are present.
1.6 Place the fish into anesthetic and monitor until breathing has slowed and swimming ceased. This should occur within less than 1-min. **Note:** Depending on the size and age of adult zebrafish, the water may have to be further titrated with Tricaine for optimal concentration.
1.7 Use a plastic spoon to transfer the fish into the Petri dish ventral side up once the fish is anesthetized.
1.8 Inject zebrafish near the ventral-midline intraperitoneally at an ~45° angle (Figure 1B). **Note:** It is critical that when piercing the skin, the needle only descends below the skin and not into the underlying organs as this will cause injury to internal organs and possible death.
1.9 With the needle in position, slowly inject the EdU solution and remove needle. **Note:** When properly injected there should be no blood observed at the injection site. Commonly a slight increase in the volume of the peritoneal cavity is observed.
1.10 Transfer the fish to fresh facility water and monitor that breathing and swimming is restored to normal. This should occur within a few minutes.
1.11 Provide a 2-h chase period (or longer if desired), before the next EdU injection.
1.12 Repeat steps 1.5–1.11 to provide animals with a second 2-h chase period of EdU. This method will provide robust labeling of all EdU-positive cells in the *S*-phase over the 4-h period prior to sacrifice.

### PART 2: brain dissection and fixation

2.1 In a small beaker, sacrifice zebrafish using an overdose of 0.4% Tricaine and ice-cold fish water (aquarium water).
2.2 Carefully dissect out the entire brain, from the olfactory bulbs to the anterior aspect of the spinal cord (Figure 1C). Take care to remove any blood, pigment or tissue adhered to the brain, as this will interfere with OPT scanning.
2.3 Transfer clean brains into an ice-cold solution of 2% paraformaldehyde (PFA; Sigma; 158127) diluted in Phosphate Buffer (pH 7.4; Figure 1D). **Caution:** PFA is carcinogenic therefore ensure to take proper handling precautions, make solution in a fume hood, and perform dissections in a well-ventilated room. PFA waste should be discarded according to institutional protocols. **Note:** Use glass vials or non-sticky plastic vials to prevent tissue from sticking. Multiple brains can be placed in a single vial for a treatment condition. Continue subsequent steps in glass vials until commencing EdU staining.
2.4 Place samples on a tissue rocker in the dark at 4°C, and rock gently overnight (8–12 h).

### PART 3: rinsing and EdU staining

3.1 Transfer samples into cooled, syringe-filtered 1X-PBS containing 0.3% Triton X-100 (Tx, Sigma; T9284) and rinse 4 × 30-min in the dark on a tissue rocker at 4°C.
3.2 Transfer brains into a solution of cooled, syringe-filtered 1% Tx/5% dimethyl sulfoxide (DMSO; Millipore; 317275) in 1X-PBS for 24-h in the dark at 4°C on a tissue rocker.
3.3 Rinse brains with cooled, syringe-filtered 1X-PBS-Tx 0.3% 4 × 30-min and leave overnight (8–12 h) on rocker in the dark at 4°C.
3.4 Prepare EdU staining solution at a volume of 3 mL/well using a 12-well plate (Corning, Inc.; 353043). The Alexa Fluor Azide (ThermoFisher) chosen to label EdU-positive cells should be either 555 (red) or 647 (far red), to reserve the 488 (green) channel for autofluorescence scans of brain volume. **Note:** No more than 5–6 brains should be placed in a single well for optimal staining (Figure 1E).

Table 1 provides the recipe for making 3 mL of staining solution (enough for a single well of a 12-well plate) from individual reagents (see supplier information in Table 2) without the need to purchase the more costly “Click-iT EdU Colorimetric IHC Detection Kit” (ThermoFisher; C10644).

3.5 Decant buffer with brain samples into a Petri dish. Using a plastic transfer pipette, carefully transfer each brain from buffer into the designated well of the 12-well plate (ThermoFisher; 150200). **Note:** Often it is necessary to cut the end of the pipette so that brains are drawn up without damage.
3.6 Using a transfer pipette, remove excess buffer from wells.
3.7 Gently add the EdU staining solution down the sides of the well and stain for 4 consecutive days with gentle agitation in the dark at 4°C.
3.8 Upon completion of staining, rinse tissue continuously with cold 1X-PBS until brains no longer show traces of Azide dye. This normally takes ½-full day of rinsing (4–8 washes).
3.9 Verify staining under a fluorescent dissecting microscope before proceeding to agarose embedding. Staining should be distinct with minimal background.

**Table 1:** Recipe for 3 mL of EdU staining solution.

**Reagent**	**Volume**
1X- PBS (pH 7.4)	2,241 μL
0.5 M L-Ascorbic acid (dissolved in Milli-Q water)	600 μL
2M Tris buffer (pH 8.5)	150 μL
100 mM Alexa Fluor Azide (dissolved in DMSO)	6 μL
1M Copper Sulfate (CuSO_4_)	3 μL
Total volume	3 mL

**Table 2:** Reagent specifications to make EdU staining solution.

**Reagent**	**Supplier**	**Item #**
L-Ascorbic acid	Sigma	A5960
Trizma base	Sigma	T6791
Copper (II) Sulfate	Sigma	C1297
Alexa Fluor 555 Azide	ThermoFisher	A20012
Alexa Fluor 647 Azide	ThermoFisher	A10277
Alexa Fluor 488 Azide	ThermoFisher	A10266

### PART 4: immunohistochemistry (*optional*)

In some instances, it may be advantageous to examine changes in cell proliferation (EdU) in association with other proteins or GFP-reporter lines. Outlined below is an exemplary protocol combining EdU labeling in the Tg(mpeg1:gfp) transgenic line that labels resident or infiltrating macrophages (Ellett et al., 2011). However, preliminary experiments should always be done to optimize any immunohistochemistry whole brain labeling for specific transgenic lines or antibodies, as difficulties in tissue penetration or quenching of fluorescence during the dehydration and/or clearing process can occur. From preliminary testing (data not shown), membrane bound reporter lines or small antibodies appear to be good candidates for whole brain labeling.

#### Whole-brain labeling of green-fluorescent protein (GFP) in the Tg(mpeg1:gfp) line

4.1 Following EdU labeling and rinsing, transfer tissue to new, clean wells of the 12-well plate to commence immunohistochemistry. All steps should be done at 4°C in the dark on a tissue agitator/rocker.
4.2 Block samples in a cooled, syringe-filtered, solution of 1X-PBS with 0.3% Tx, 2% normal goat serum (Sigma; G9023), and 1% bovine serum albumin (Sigma; A7906) for 8-h.
4.3 Incubate brains in the primary antibody, rabbit-anti-GFP (ThermoFisher; A11122) diluted 1:500 with blocking solution (above) for 7-days.
4.4 Rinse samples in 1X-PBS-Tx 0.3% for 4 × 1-h.
4.5 Incubate brains in the secondary antibody, goat-anti-rabbit Alexa 555 (ThermoFisher; A21429) diluted 1:750 with 1X-PBS-Tx 0.3% for 7-days.
4.6 Rinse samples in 1X-PBS-Tx 0.3% for 4 × 1-h.
4.7 Verify staining under a fluorescent dissecting microscope before proceeding to agarose embedding. Staining should be distinct with minimal background throughout the brain tissue (dorsal or cross-sectional view) if successful.

### PART 5: tissue embedding

5.1 Prior to embedding, wash samples with double distilled Milli-Q water, 3 × 30-min to remove any excess salt from 1X-PBS rinses.
5.2 Combine low melting agarose (Sigma; A9414) with water to yield a 1% solution (Combes et al., 2014). Typically, 0.25 g/25 ml is sufficient for a single well of a 6-well plate (Corning, Inc.; 353046). Heat solution gradually in a flask using a microwave until dissolved. **Caution:** Solution will be extremely hot once dissolved, therefore use proper handling equipment.
5.3 Cool agarose solution under tap water until flask can be comfortably touched to wrist.
5.4 Once cooled, pour solution into a 50 mL Luer lock syringe with a 0.45 μm syringe filter membrane attached and filter solution into wells. Fill each well just below the brim.
5.5 Monitor the temperature of agarose in wells with a thermometer. Temperature must reach ~30°C or below before brains are transferred. **Note:** Use a bed of ice to speed up cooling process. Be sure that the temperature does not drop much below 30°C or brain samples cannot be properly oriented as agarose will start solidifying.
5.6 Insert each brain into a single well using a cut plastic transfer pipette or cut pipette tip of a 1,000 μL micropipette. Brains should be placed down one side of the well with as little buffer as possible.
5.7 Use long fire-polished glass Pasteur pipettes (or other) to manipulate each brain sample and orient dorsal side up. **Note:** The goal is to situate samples in the middle of the well (i.e., between top and bottom and in the center; Figure 1F). For best imaging tissue should be embedded along the long-axis. For adult zebrafish brains this means that the A-P neuro-axis is oriented vertically when the block is standing up and when mounted for scanning. This method provides less variability in the depth of tissue through which light must pass as the sample is being imaged around 360°.
5.8 Allow agarose to set for a minimum of 1-h at 4°C in the dark (Figure 1F). **Note:** The protocol can be paused here.

### PART 6: trimming

6.1 Use the back of a scalpel blade to cut around the agarose cylinder and place it onto a clean glass or plastic surface for trimming (Figure 1G).
6.2 With the agarose cylinder in the same orientation as in the 6-well plate (i.e., brain dorsal side up), use a razor blade to make 4 initial cuts to form a trapezoid (Figure 1H, labels 1–4).
  1. The short side of the trapezoid should be made 1 cm from the anterior aspect of the olfactory bulbs, by making a straight cut perpendicular to the bulbs.
  2. The long side of the trapezoid should be made at least 1.5 cm from the posterior aspect of the spinal cord, cutting perpendicular to brain axis.
  3&4. Make a single diagonal cut on the lateral sides of the brain to join cuts 1 and 2.

6.3 Use a scalpel blade to gently orient the block so that the olfactory bulbs are up (i.e., brain should be oriented vertically along the long-axis; Figure 1I).
6.4 Next use a clean scalpel blade to trim the block. Start by trimming vertically down each of the four corners.
6.5 Continue around the block until there is ~5 mm of agarose around the brain and the block tapers to a base of ~1 cm (size of mount face to which block is glued; Figure 1J). The goal is to have an equal amount of agarose around the entire brain for consistent penetration of light during imaging. Ensure the height of the block base (i.e., distance from tip of spinal cord and bottom of block) is no < 1 cm when finished, as less than this could lead to the glue interfering with OPT imaging.
6.6 Using a brightfield microscope, verify the orientation of the brain within the trimmed agarose block along the long-axis (Figure 1K) and in the vertical plane (Figure 1L). **Note:** The protocol can be paused here with samples stored at 4°C in the dark.

### PART 7: dehydration

7.1 Transfer trimmed blocks into labeled 50 mL Falcon tubes. Place no more than 4 samples/tube. **Caution:** Perform all methanol dehydration steps in a fume hood taking proper safety measures. Methanol waste should be discarded according to institutional protocols.
7.2 For a single block, fill tube with 25 mL of 100% HPLC grade methanol to dehydrate tissue. If preparing multiple blocks fill to 50 mL. The same Falcon tubes can be used for all subsequent dehydration and clearing steps. **Note:** Label tubes well with a non-removal marker (i.e., china marker), since labels can be easily lost by methanol or BABB exposure.
7.3 Place tubes in the dark at room temperature on a tissue rocker for 4-h. **Note:** To prevent any solution leaking onto the tissue rocker, place the tubes into a secondary plastic container.
7.4 Repeat step 7.2–7.3 with 3 additional methanol changes. If left overnight, this is still considered a single methanol rinse. **Note:** To check if dehydration was successful, take a couple of milliliters of methanol from tissue rinse and mix with BABB in a glass petri dish. If color turns cloudy once mixed tissue is not fully dehydrated and water is left in sample. Consider a further methanol rinse to remove remaining water.

### PART 8: clearing

8.1 Prepare a 2:1 solution of fresh benzyl benzoate: benzyl alcohol (BABB; Sigma: B6630, 402834). **Caution**: BABB is considered hazardous and thus proper safety measures must be taken. Work in the fume hood when making solution and for all subsequent solution changes. BABB solution should be stored out of direct light, and waste discarded according to institutional protocols.
8.2 Using the same tubes as for methanol dehydration, place samples in the first BABB rinse. If previously used BABB is available, use this for the first BABB rinse. Otherwise, use new BABB. **Note:** Previously used BABB can only be used for the first rinse. Thereafter, fresh BABB must be used for proper clearing.
8.3 Place tubes in the dark at room temperature on a tissue rocker for 4-h. **Note:** To prevent any solution leaking onto the tissue rocker, maintain tubes in the secondary plastic container.
8.4 Repeat steps 8.2–8.3 with 3 additional BABB changes. If left overnight, this is still considered a single BABB rinse.
8.5 Upon completion of BABB rinses, verify that the expected staining pattern is observed under a fluorescent dissecting microscope before proceeding to OPT scanning. **Note:** Properly cleared brain samples should appear nearly transparent *en block* under bright light compared to before dehydration and clearing steps (Figure 1M).
8.6 Samples should be imaged using OPT within 1–2 days of completion of the above protocol to prevent any fading of fluorescent signal and minimize exposure time during scans.

### Comments on optical projection tomography (OPT) scanning and post-processing data reconstructions for downstream visualization and analysis

The detailed methods of use of the Bioptonics 3001 OPT scanner (Bioptonics, Edinburgh, UK) and Nrecon reconstruction software (Bruker microCT) is beyond the scope of the presented protocol. Nonetheless, below are listed some general guidelines and considerations for scanning cleared adult zebrafish brain tissue, with example parameters of an OPT scan shown in Table 3.

**Table 3:** Example parameters for OPT scanning of EdU and GFP in the Tg(mpeg1:gfp) transgenic line of a 6-month adult zebrafish brain.

**Visualization**	**Staining procedure**	**OPT laser**	**Exposure (ms)**	**Averaging**	**Rotation**	**Scan time**
EdU	Alexa Fluor 647 Azide	647	~200	2	0.45°	~1-h
GFP	1° Rabbit-anti-GFP (1:500)	555	~150	2	0.45°	~1-h
	2° Goat-anti-rabbit-Alexa555 (1:750)					
Brain volume	none	488	~800	None	0.45°	~40-min

#### OPT scanning:

1. Provide 20–30 min for blocks to adhere to the OPT mount if using an adhesive (Figure 1N). Superglues such as Lock tite and Tarzan grip work well and can be purchased from local suppliers. Note that the types of OPT mounts can vary, and the use of glues and how the block is initially trimmed may require modification.
2. Ensure the BABB in the OPT chamber in which samples are submerged is always clean, as are the sides of the glass chamber.
3. Time should be taken to always properly calibrate the OPT scanner before the first experimental sample is imaged.
4. It is advisable to standardize the magnification at which all samples are scanned.
5. Ensure each channel (i.e., 350, 488, 555, 647) is in focus and that exposure is adjusted so that no “bright spots” are seen on your sample—this will affect post-processing reconstructions. For instance, use a general rule of keeping the exposure for each channel at ~75% of the maximum, but this must be assessed on a case by case basis. Similar to conventional confocal microscopy, the staining intensity of EdU (or other markers) will vary slightly from sample to sample. However, if downstream analysis is to compare intensity levels under different conditions, appropriate preliminary experiments should be performed to determine consistent parameters for scanning.
6. For scanning, image at least every 0.45° around the 360° axis of the sample. Modifications of this can readily be done, but will influence the length of time to scan a single sample.
7. Averaging can also be applied to each individual channel. When exposure is typically lower than 500 ms, averaging may be advantageous.
8. Upon completion of each scan, use a Data Viewer to review that all frames in the data series were imaged properly before removing sample from the OPT scanner.
9. Samples can be stored in BABB in the dark at room temperature or 4°C for a couple months with EdU fluorescence remaining fairly stable.

#### Post-processing data reconstructions:

1. Reconstruction is done by using the raw OPT files produced from OPT scans.
2. Prior to commencing reconstruction, transfer all raw OPT files onto the PC that houses the reconstruction software. The raw OPT files/channel will have a file size of ~1.5 GB. Following reconstruction, each dataset/channel will increase to >6 GB so be certain to have sufficient hard drive space on the PC.
3. While processing the raw OPT files to yield a final reconstructed dataset for each individual channel, ensure that the final reconstruction is crisp and in focus. Final datasets should be in focus when viewed either in 3-dimensions (3-D) or in cross-sectional view. If blurry/fussy, adjust parameters for reconstruction and re-run, or omit from downstream analysis if it remains out of focus.
4. Reconstructed datasets can be loaded into software such as Drishti, IMARIS, or FIJI/IMAGEJ for visualization and quantification. Depending on the settings used during OPT scanning, differences in staining intensity, total volume, or surface area of a marker can be reliably quantified between treatment groups. From experience, for 3-D visualization of single or multiple OPT channels, IMARIS is optimal. However, both IMARIS and FIJI/IMAGEJ provide different quantification methods (2-D or 3-D).

### Telencephalic stab lesion assay

Adult fish were anesthetized in 0.04% Tricaine (Sigma) in fish water prior to stab lesion. Stab lesions were performed as described in Kroehne et al. (2011). In brief, a 30 gauge cannula was inserted through a single nostril along the rostrocaudal brain-axis, into the olfactory bulb and finally the caudal aspect of the telencephalic hemisphere. Thereafter, fish were returned to their experimental tank and monitored for normal swimming behavior.

### Quantification of whole brain EdU stained tissue

All quantification methods described below were developed and performed using FIJI/IMAGEJ software to investigate systemic, brain-wide changes in EdU labeling following adult telencephalic stab lesion. Original OPT scans were taken at a screen resolution of 1,024 pixels. Analysis methods relied on 16-bit grayscale pixel intensity of EdU in the region of interest (ROI). Pixel intensity ranged from 0 to 65,536 (0 = black; 65,536 = white). Preliminary experiments were performed to standardized the exposure for channels during OPT scans between uninjured and injured brains for downstream analysis. Virtual cross-sections were a depth of 1-pixel each following post-processing and 3-D reconstruction of brains, resulting in 1,024 slices through the A-P brain axis.

#### Histogram analysis

To examine changes in cell proliferation across the A-P neuro-axis of the adult zebrafish brain under homeostasis and following lesion, we developed an analysis method allowing us to plot the histogram of EdU intensity (**Figure 4A**). 3-D reconstructed, EdU-stained brains were virtually sectioned through the horizontal plane, and a final maximum projection obtained. A threshold mask, was next overlapped on all non-black pixels only and the brain segmented along its A-P axis into individual 20 μm segments. Since all animals were age-matched, a near equal number of segments was derived from each brain for comparison. From each segment, the mean pixel intensity was calculated as a representation of the mean EdU intensity within the segment. The mean EdU intensity per like-segment was then calculated across biological samples and plotted as a histogram for control and lesioned conditions or as the percent change from control levels.

#### Structure analysis

To quantify systemic changes in the amount of EdU-positive staining in structures of the lesioned brain compared to control, we developed an analysis method targeted to large tissue depths (**Figure 5A**). ROI's in structures across the brain axis, both proximal and distal to the site of lesion were analyzed, however, only a subset of the data is displayed. For each ROI, the pixel depth in the z-axis was determined, and then converted to 16-color pixel bins. The 16-color pixel bins divided the 65,536 intensity range of pixels into bins of 4,096 each, designated by progressively warmer colors. We then calculated the total number of pixels within each bin and converted pixels into voxels. Pixel size ranged between ~4.5 and 5 μm, with associated voxel size ~91–125 μm^3^ across brain samples. The underlying hypothesis was that more non-black pixels should be observed in cases where more EdU labeling was present. This approach provided us with 15 non-black bins for downstream analysis of changes in EdU volume within a defined brain structure. For individual biological samples, the pixel count was summed across the 15 bins and converted to volume (μm^3^). The mean volume was then calculated across biological samples in the same ROI, upon which statistical analysis was performed.

#### Slice analysis

To reliably quantify changes in EdU-positive labeling in distinct adult stem cell niches throughout the brain post-injury, we devised a slice analysis method that sampled EdU volume every 5th section along the z-axis of the neurogenic compartment (**Figure 6A**). This method was developed to avoid EdU labeling of immune cells (i.e., neutrophils, macrophages) that are activated post-lesion and recruited near the site of injury. Following selection of the ROI's for analysis and determination of its z-depth in pixels, in every 5th section the stem cell niche was demarcated by hashed lines, converted to 16-color pixel bins as previous, and the total number of pixels/bin extracted. We next summed all pixels across slices in subsets of non-black bins (e.g., 4,096–12,288) of each biological sample. These values were then converted to voxels to represent EdU volume and the final mean calculated across biological replicates for statistical comparisons.

### Larval EdU labeling and imaging

Larvae (Tubingen) were housed according to standard protocols. Larvae were transferred into a 50 ng/uL EdU solution with 1% dimethyl sulfoxide (DMSO) in 1x Ringer's media and incubated for 12-h at 28.5°C. At 3-, 5-, and 7-dpf (days post fertilization) animals were sacrificed by an overdose of 0.04% Tricaine, then immediately fixed overnight in ice-cold 4% PFA in 0.1 M phosphate buffer (pH 7.4). Following 1X-PBS rinses, the dorsocranial skin overlaying the brain was removed from larvae for subsequent EdU labeling. Samples were incubated in EdU staining solution as described above for 30-min in the dark at room temperature with gentle rocking. Thereafter, larvae were rinsed then incubated for 30-min in a 1:5,000 solution of 4,6-Diamidino-2-phenylindole (DAPI) for counterstaining. Brains were next excised, washed, and placed through an ascending glycerol series (30%
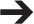
50%
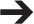
70%), before finally being whole-mounted for confocal imaging in 70% glycerol. Samples were imaged using a Leica TCS SP8 inverted confocal laser scanning microscope equipped with a Leica HyD hybrid detector. Acquisition was performed in 1 μm z-steps using a 20X oil-immersion objective at 1,024 resolution. Acquired z-stacks were visualized in 3-D using IMARIS software.

### Statistical analysis and data representation

Statistical analyses and data representation were completed using GraphPad Prism 7. One-way ANOVA was used to compare differences in EdU-positive labeling between the control group and 1-, 3-, and 7-day post lesion (dpl). Where a significant difference was reported, Tukey's *post-hoc* test was applied with significance accepted at *p* < 0.05. All data are represented graphically as either EdU intensity (histogram analysis) or EdU volume (μm^3^; structure and slice analysis). All data shown represent mean + standard error of the mean (S.E.M.).

## Results

### EdU labeling is successfully visualized throughout the 3-dimensional axis of the adult brain

**Figure 2:**
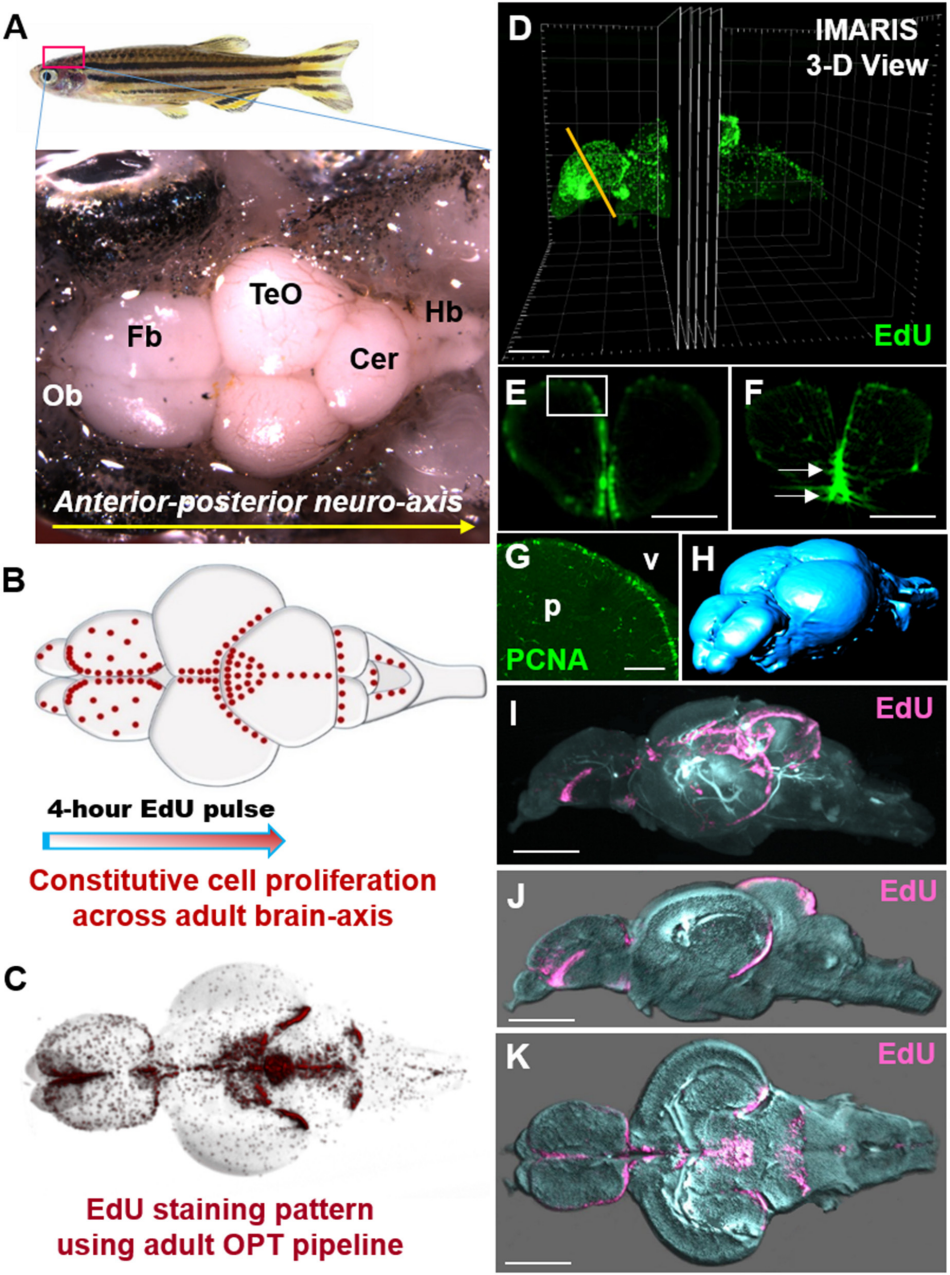
Whole brain EdU labeling and 3-dimensional OPT scanning recapitulates the constitutive pattern of cell proliferation in the adult zebrafish brains. **(A)** Dorsal view of adult zebrafish brain displaying major structures along the A-P neuro-axis. Ob, olfactory bulbs; Fb, forebrain; TeO, optic tectum; Cer, cerebellum; Hb, hindbrain. **(B)** Schematic dorsal view of adult brain showing the known constitutive pattern (Kaslin et al., 2008) of cell proliferation (red dots) along the brain axis following a 4-h EdU chase. **(C)** Dorsal view of EdU staining using our adult OPT pipeline demonstrating the same labeling pattern across the brain axis as in **(B). (D)** Example of IMARIS 3-D visualization output of an adult EdU injected brain (green) illustrating the ability to visualize or analyse regions of interest in cross-section (or other planes). Yellow line depicts level of telencephalic cross-section shown in **(E,F). (E,F)** Cross-sections through the adult zebrafish telencephalon showing examples of optimal (**E**; near cellular resolution) and suboptimal **(F)** EdU staining (green)/OPT imaging along the periventricular neurogenic niche following data reconstructions. White box in **(E)** denotes dorsal telencephalic domain shown in **(G)**. White arrows in **(F)** show EdU that was over-exposed during scanning, while the slightly fussy image indicates that the post-processing software reconstruction was of poor quality. **(G)** Antibody labeling using Proliferating Cell Nuclear Antigen (PCNA) displaying the homeostatic pattern of cell proliferation at the dorsal telencephalon from cryosectioned, confocal-imaged tissue. Note that labeling is restricted to the stem cell niche adjacent the forebrain ventricle (v) with little to no staining within the parenchyma (p). **(H)** Anterior-dorsal view of iso-surface rendered adult brain (blue) using IMARIS software derived from initial OPT autofluorescence scans of brain contour. **(I–K)** Constitutive brain EdU labeling (pink) across the neuro-axis merged with an autofluorescence scan of brain morphology/volume (pale blue) shown in mid-sagittal **(I,J)** and horizontal **(K)** views. In **(J,K)** images were rendered in IMARIS. Scale bars: **(E,F)** = 300 μm; **(G)** = 150 μm; **(D,I–K)** = 500 μm.

The protocol outlined here successfully demonstrates the ability to label proliferating cells in the whole adult zebrafish brain with only a short 4-h pulse of the *S*-phase marker, 5-ethynyl-2′-deoxyuridine (EdU; Figures 2A–C). OPT scans of EdU injected adult fish show consistency of labeling across constitutively proliferating stem cell populations residing in adult neurogenic niches along the neuro-axis (Figures 2B,C; Supplementary Video 2). Combining this sample preparation with isotropic imaging by OPT permits near cellular resolution of actively cycling cells that can be visualized in 3-dimensions or by section for analysis (Figure 2D). The intense labeling of EdU primarily observed along the midline of the brain where adult stem cell niches reside, closely mimics the proliferative pattern previously shown by standard immunohistochemistry performed on cryosections (Lindsey and Kaslin, 2017). For instance, the specificity of EdU labeling in the dorsal forebrain adjacent the ventricle in virtual cross-sections from our OPT pipeline are consistent with immunolabeling using the common proliferative marker, Proliferating Cell Nuclear Antigen (PCNA) in this same domain (compare Figure 2E inset with Figure 2G). However, suboptimal EdU labeling and OPT scanning can result in sections that are over-exposed, such as shown in Figure 2F (white arrows) in the forebrain ventral midline, and give rise to poor reconstruction post-OPT imaging. Output such as this considerably impairs analysis of EdU using either intensity or volumetric analyses, and therefore such samples should be re-examined or omitted. Since OPT scanning is performed using 1,024 pixels, following reconstruction brains stacks have a depth of 1-pixel each. When considering downstream analyses, it is important to visualize datasets in cross-section to confirm the expected staining pattern, assess for over-exposure and that reconstructions were completed properly. This can be verified immediately using dataset reconstruction software (i.e., NRecon) or using programs such as IMARIS. Additionally, by taking advantage of the autofluorescence of brain tissue, OPT scanning in a channel not reserved for a specific marker (here the 488 laser), allows users to obtain the volume of the brain (or shell) that can be visualized independently for morphometric analysis (Figure 2H) or overlaid with the EdU-specific channel using IMARIS software (Figures 2I–K). These samples can be visualized in the plane of choice from either the 3-D reconstructed (Figure 2I) or rendered (Figures 2J,K; Supplementary Video 3) dataset. Merging scans taken of the brain shell with EdU scans is valuable to define the neuro-anatomical localization of EdU patterns under healthy or diseases states.

### Immunohistochemistry can be performed on EdU-labeled brains but optimization is critical

**Figure 3:**
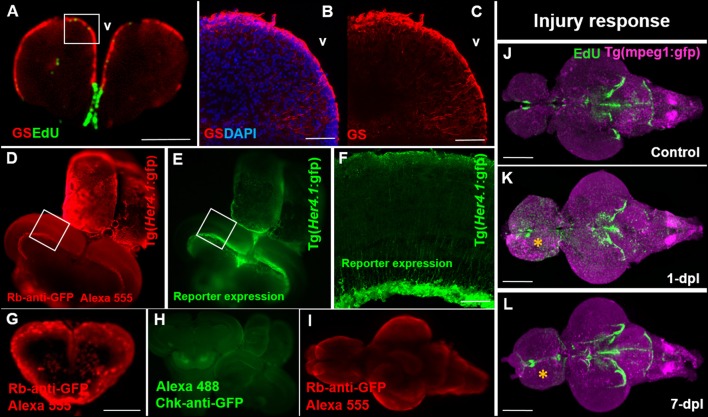
Compatibility of immunohistochemistry with OPT pipeline. **(A)** Successful double-labeling and OPT scanning of EdU (green) and the glial marker, glutamine synthetase (GS; red) in the adult zebrafish forebrain. White box denotes images shown in **(B,C)**. **(B,C)** Co-labeling with DAPI (blue; **B**) and single **(C)** antibody labeling of GS (red) in cryosectioned, confocal-imaged tissue confirming the specificity of GS labeling shown using our OPT pipeline. **(D,E)** Whole brain immunohistochemistry in the adult zebrafish using a rabbit-anti-GFP primary antibody conjugated to Alexa 555 **(D)** recapitulates the same staining pattern seen with the endogenous GFP reporter in the Tg(*Her4.1*:gfp) transgenic line **(E)** prior to dehydration and clearing. White boxes in **(D,E)** depicts location of image displayed in **(F)**. **(F)** GFP-positive staining in the deep quiescent glial layer of the periventricular gray zone of the adult optic tectum shown in cryosectioned, confocal-imaged tissue in the Tg(*Her4.1*:gfp) line mimicking the GFP pattern seen in tissue prepared using our OPT pipeline. **(G–I)** Examples of poor antibody penetration **(G)**, poor immuno-labeling, and **(I)** quenched antibody staining post-OPT imaging using different GFP antibodies. **(J–L)** Adult control brain **(J)** in the Tg(mpeg1:gfp) macrophage line injected with EdU compared with brains post telencephalic lesion (yellow symbol) examined at 1-day post lesion (dpl; **K**) and 7-dpl **(L)** for changes in macrophage distribution (purple) and cell proliferation (green). Scale bars: **A, G** = 300 μm; **B-C** = 150 μm; **F** = 200 μm; **J-K** = 500 μm.

The ability to observe cell-specific reporter lines or proteins of interest throughout the adult brain alongside proliferative patterns is beneficial to explore interactions between different cell types or changes in the proliferative status of a given population. While a secondary focus of our study and an optional step in the current protocol, presented here are some examples of successful immunostaining accomplished at different stages of the EdU sample preparation pipeline. It is strongly encouraged that optimization of each individual transgenic line or protein is completed prior to implementation with this protocol. Immunohistochemistry (IHC) labeling is performed post-EdU staining. Brains require 1-week incubation in primary and secondary antibodies for reasonable fluorescent labeling through the entire depth of the adult brain. We demonstrate that co-labeling of EdU with the commonly used glial marker, glutamine synthetase (GS), can be accomplished using our OPT pipeline (Figure 3A) and that this antibody displays the same labeling pattern observed in cryosections (Figures 3B,C). In cases where GFP reporter lines are utilized it is important that antibody labeling (Figure 3D) parallels the endogenous pattern of GFP reporter expression (Figure 3E) during sample preparation for OPT scanning, and resembles GFP reporter expression in sectioned tissue (Figure 3F). It is advisable to verify IHC staining patterns prior to sample embedding, dehydration, and clearing, by cutting through the thickest section of the brain after the 2-week incubation, and in a region where the pattern of staining is well-known. It is not uncommon for some primary antibodies to work well and others poorly in whole brain IHC (Figure 3G). Most critical however, is that the fluorescent signal is maintained following the dehydration and clearing steps to allow successful OPT imaging of fluorescent channels. Poor antibody labeling during whole brain staining may be a consequence of many factors, but often can be visually detected by limited penetration into the parenchyma of tissue (Figure 3G), unsuccessful labeling of an antibody (Figure 3H), or quenching from the dehydration and clearing process (Figure 3I). Nonetheless, when IHC labeling protocols are optimized for transgenic lines in combination with EdU labeling, it is possible to readily observe systemic changes in distinct cell populations of interest. For example, performing whole brain EdU labeling in the Tg(mpeg1:gfp) transgenic line specific to tissue macrophages under homeostasis and post-telencephalic injury, one can detect a global increase in the intensity of both EdU and immune cell staining at 1-dpl throughout the lesioned hemisphere (yellow asterisk) that returns near baseline by 7-dpl (Figures 3J–L; dpl, days post lesion).

### Reconstructed OPT datasets can easily be analyzed using FIJI/IMAGEJ software

**Figure 4:**
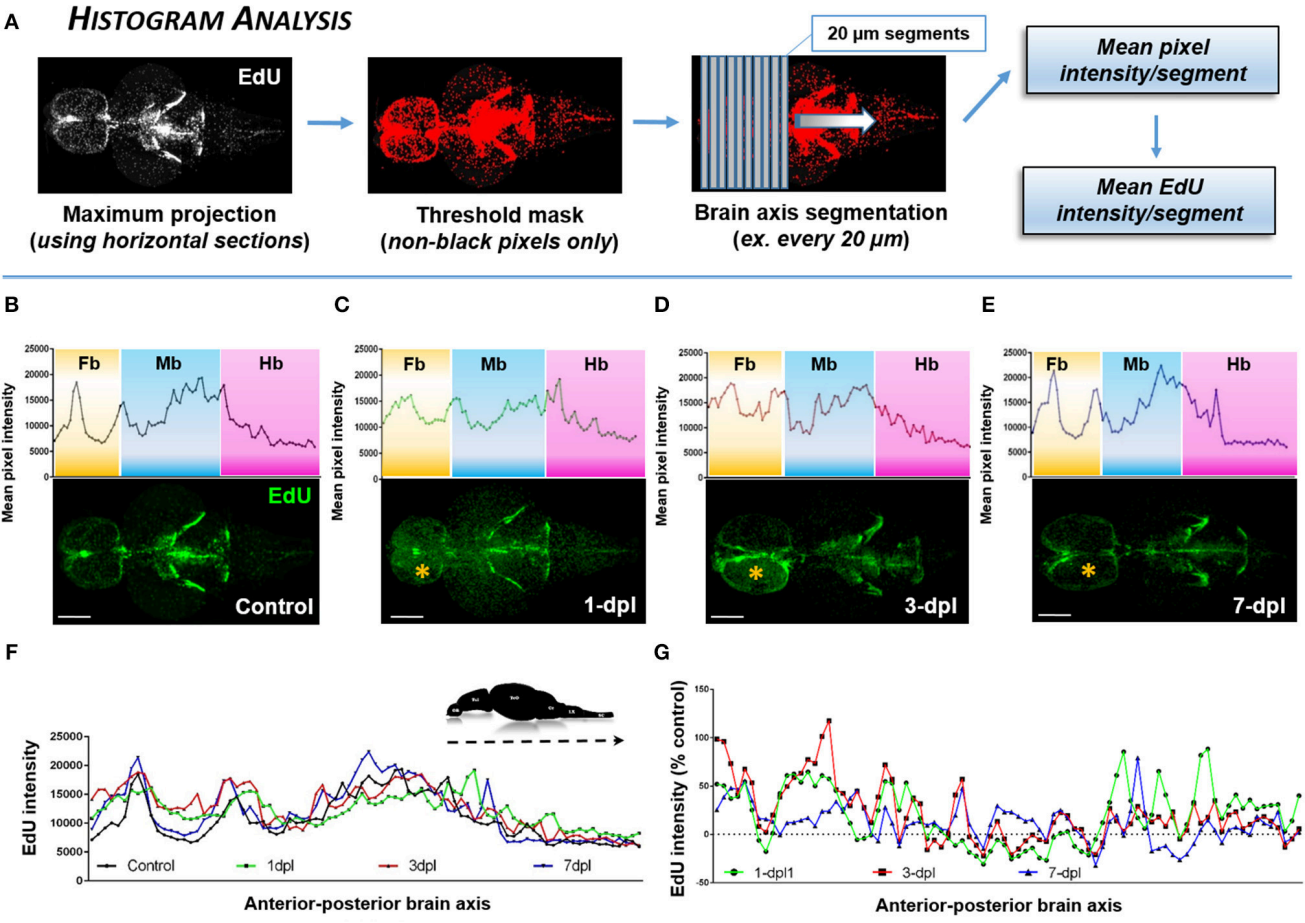
Identifying the proliferative profile across the brain axis following injury using *Histogram Analysis*. **(A)** *Histogram Analysis* workflow using FIJI/IMAGEJ showing a maximum projection derived from horizontal sections and overlayed with a threshold mask to detect only non-black pixels. By segmenting the brain along the A-P axis the mean pixel intensity/segment can be used to represent the mean EdU intensity/segment. **(B–E)** Mean pixel intensity of EdU per segment plotted as a histogram across the brain axis displaying conspicuous peaks in brain regions where greater EdU labeling (green) is present shown in control (**B**; *n* = 5 brains) and 1-dpl (**C**; *n* = 4 brains), 3-dpl (**D**; *n* = 4 brains), and 7-dpl (**E**; *n* = 5 brains) treated animals. The yellow asterisk denotes the lesioned telencephalic hemisphere. Fb, forebrain; Mb, midbrain; Hb, hindbrain. **(F,G)** EdU intensity plotted across the A-P brain axis compared across all groups **(F)** and normalized as the percent change from control **(G)**. Scale bars: **(B–E)** = 500 μm.

Numerous methods of quantification can be used to analyze OPT datasets. Here, we present three analysis methods we developed and performed using FIJI/IMAGEJ from reconstructed OPT datasets to interrogate changes in EdU intensity or volume across the neuro-axis and within distinct structures or proliferation zones of the brain. By using non-black pixel intensity as a direct proxy of EdU intensity along the A-P axis of the adult brain (Figure 4A), our *Histogram Analysis* results demonstrate the ability to create a baseline/reference profile of cell proliferation and to monitor how this profile is perturbed following forebrain telencephalic lesion (Figures 4B–G). For example, during constitutive cell proliferation peaks in EdU intensity are closely associated with adult proliferative zones along the ventricular system (Figure 4B). One of the most conspicuous peaks in EdU intensity is observed in the midline forebrain (Fb) niche (proliferation zones 2 & 3 in Grandel et al., 2006), while little staining is observed elsewhere in either telencephalic hemispheres. In the midbrain (Mb) and continuing into the hindbrain (Hb), the observed EdU peaks are a product of a collection of well documented proliferation zones (proliferation zones 7–14). The Mb bin primarily represent proliferation in the hypothalamus, tectum, torus semicircularis and posterior mesencephalic lamina (10–13). The Hb bin primarily represent proliferation in the cerebellum (14). Tracking the forebrain profile of EdU intensity over the first week post-injury shows that at 1-dpl the overall pattern of EdU intensity is elevated along the A-P axis of the forebrain, in particular in the forebrain parenchyma, likely as a consequence of proliferating immune cells (i.e., macrophages, neutrophils) recruited to the site of lesion (Figure 4C). Moreover, the EdU profile across all brain segments (i.e., individual points plotted on histograms) of the A-P axis at 1-dpl appear changed as a result of the cumulative difference in EdU intensity staining per segment with injury compared to control levels. By 3-dpl EdU intensity is restricted largely to the lesioned hemisphere (Figure 4D; yellow asterisk), whereby at 7-dpl the histogram of forebrain EdU intensity closely resembles the constitutive profile (Figure 4E). By plotting all EdU histograms (control, 1-, 3-, 7-dpl) together (Figure 4F) or representing the data as the percent change from control (Figure 4G), we further show the ability to compared major peaks in cell proliferation for statistical analysis across treatments within a defined range of A-P brain segments of interest using our *Histogram Analysis*.

**Figure 5:**
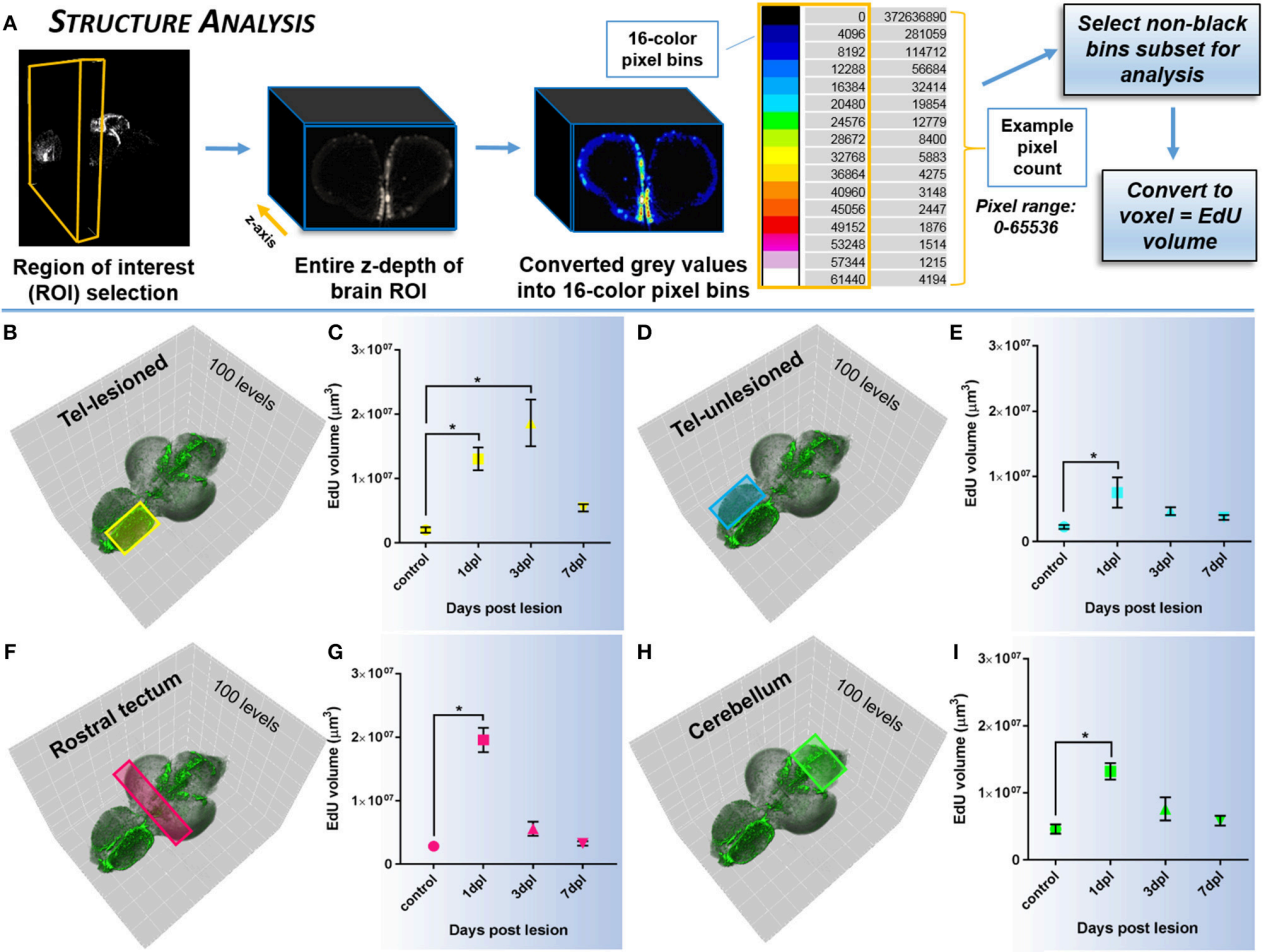
Investigating systemic changes in cell proliferation within major brain subdivisions following brain injury using *Structure Analysis*. **(A)** *Structure Analysis* workflow using FIJI/IMAGEJ showing z-depth of brain region of interest converted from grayscale to 16-color pixel bins to obtain pixel counts/non-black bins for final analysis of EdU volume in voxels. Cross-sectional view shown is from the forebrain telencephalon. The 16-color pixel bins are arranged from cooler to warmer colors, indicating greater pixel intensity values at the upper end of the pixel range. **(B–I)** Proliferative response across four major adult brain structures compared to control in the adult zebrafish brain at 3 time-points (1, 3, 7-dpl) following telencephalic lesion. Brain structures analyzed are indicated by colored rectangles overlayed on 3-D rendered adult zebrafish brains at 3-dpl (EdU, green). For all structures a total of 100 pixel levels through the A-P axis were used for quantification, with *Structure Analysis* performed on all 15 non-black pixel bins. **(B,C)** Lesioned telencephalic hemisphere (yellow; *n* = 5–10 brains/group) displaying a significant increase in EdU volume compared to control at 1-dpl and 3-dpl. **(D–I)** Unlesioned telencephalic hemisphere (**D,E**; blue; *n* = 6–10 brains/group), rostral tectum (**F,G**; pink; *n* = 4–10 brains/group), and cerebellum (**H,I**, green; *n* = 4–10 brains/group) showing a significant increase in EdU volume from control at 1-dpl. ^*^Significance was accepted at *p* < 0.05; One-way ANOVA, Tukey's *post-hoc* test for multiple comparisons.

**Figure 6:**
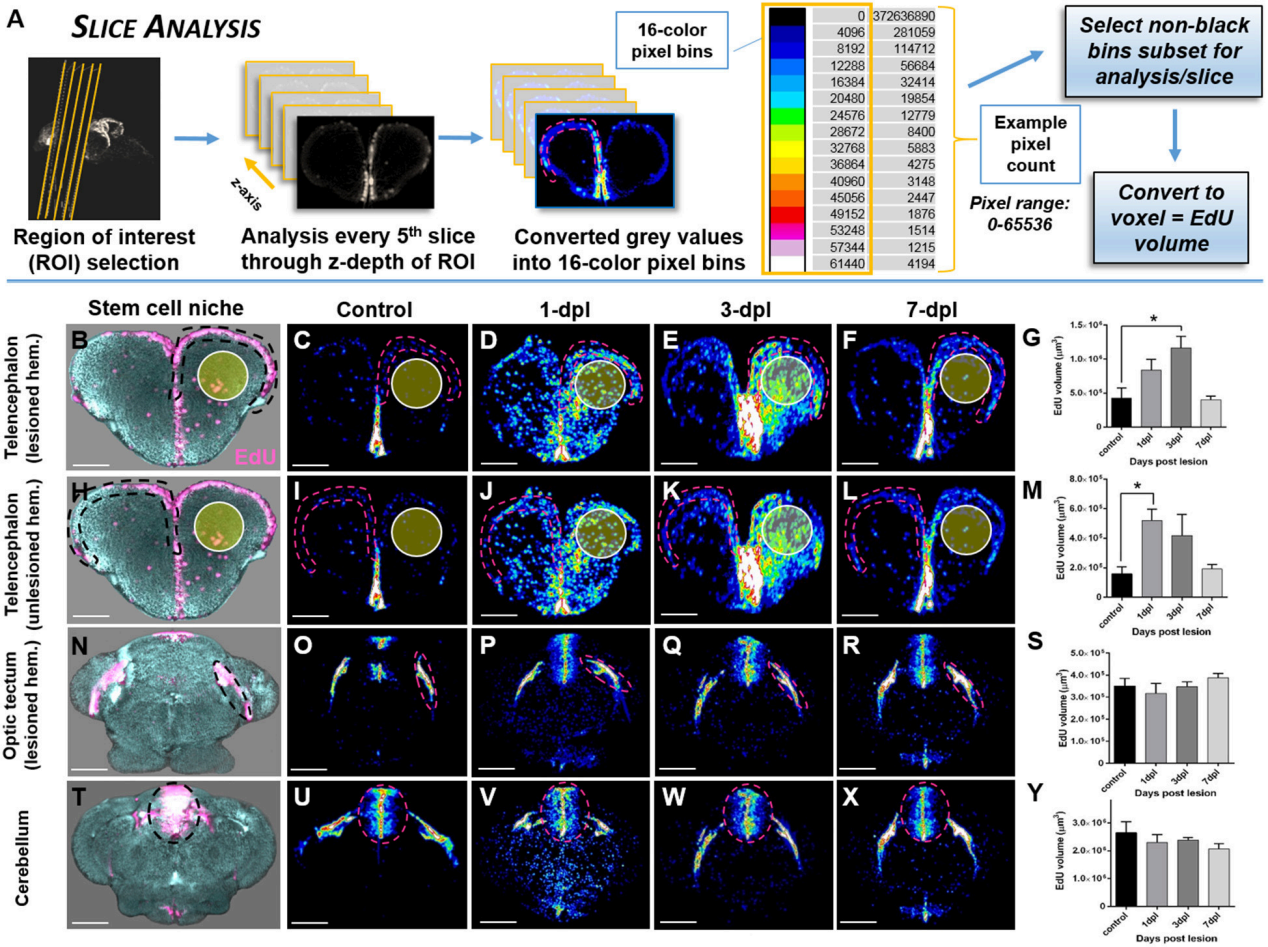
Investigating systemic changes in adult stem cell niche proliferation following brain injury using OPT *Slice Analysis*. **(A)** *Slice Analysis* workflow using FIJI/IMAGEJ showing every 5th pixel slice converted from grayscale to 16-color pixel bins to obtain pixel counts/non-black bins for final analysis of EdU volume in voxels. Cross-sections shown are from the forebrain telencephalon, with pink hashed lines denoting an example sub-region for analysis. The 16-color pixel bins are arranged from cooler to warmer colors, indicating greater pixel intensity values at the upper end of the pixel range. **(B–Y)** Proliferative response across four major adult stem cell niches compared to control in the adult zebrafish brain at 3 time-points (1, 3, 7-dpl) following telencephalic lesion. The site of lesion is denoted by the yellow circle. Hashed lines demarcate the stem cell niche quantified using *Slice Analysis*. **(B,H,N,T)** Representative 3-D rendered images from OPT datasets displaying stem cell niches denoted by EdU staining (pink). All other image panels show maximum projections of representative cross-sections converted to 16-color (FIJI look up table, LUT) for analysis of control and lesioned treatments. For analysis, pixel counts derived from only the first 3 non-black bins were used (i.e., 4,096, 8,192, 12,288). **(B–G)** Lesioned hemisphere (ipsilateral; *n* = 5–8 brains/group) of the pallial stem cell niche showing a significant increase in EdU volume from control at 3-dpl. **(H-M)** Unlesioned (contralateral; *n* = 5–8 brains/group) hemisphere of the pallial stem cell niche showing a significant increase in EdU volume from control at 1-dpl. **(N–S,T–Y)** Both tectal (**N–S**; *n* = 4–10 brains/group) and cerebellar (**T–Y**; *n* = 4–7 brains/group) stem cell niches situated more posterior to the site of injury revealed no significant difference at any of time-points post-lesion. ^*^Significance was accepted at *p* < 0.05; One-way ANOVA, Tukey's *post-hoc* test for multiple comparisons. Scale bars: **(B–F,H–L,N–R,T–X)** = 150 μm.

While quantifying the global pattern of change in EdU across the A-P axis (*Histogram Analysis*) is informative to detect the primary domains of the CNS that respond with damage, more specifically examining the systemic effect of EdU in distinct neuroanatomical structures (Structure Analysis) or niches (Slice Analysis) provides more precise data of how an individual region is modulated. By designing two quantification methods that rely on the voxel size of non-black pixels, our results reveal that using voxel size as a direct readout of EdU volume is a statistically reliable method to compare changes in regions of interest (ROI) between control and lesioned adult brains. Using *Structure Analysis* (Figure 5A), where the EdU volume within the z-depth (i.e., multiple slices) of a ROI is analyzed we show that similar to our previous findings in sectioned tissue (Kroehne et al., 2011), a statistical increase in EdU cell proliferation is present at 1-dpl and 3-dpl compared to control in the lesioned hemisphere (Figures 5B,C; One-way ANOVA; tukey's *post-hoc* tests for multiple comparisons; *p* < 0.05). Furthermore, we demonstrate for the first time that a similar statistical increase in cell division is present at 1-dpl in the unlesioned telencephalic hemisphere (Figures 5D,E), rostral tectum of the midbrain (Figures 5F,G), and hindbrain cerebellum (Figures 5H,I; One-way ANOVA; tukey's *post-hoc* tests for multiple comparisons; *p* < 0.05), indicating that lesion-induced signals are far-reaching throughout the brain axis and promote structure specific changes in cell cycle kinetics. However, applying the same volumetric EdU quantification in 1-pixel thick slice intervals in defined proliferative/neurogenic zones (pink hashed lines) along the A-P axis of the brain using our *Slice Analysis* (Figure 6A) exhibited variation in EdU volume within stem cell niches residing in the same structures analyzed previously. We reported significant increases in EdU volume at 3-dpl and 1-dpl in the lesioned (Figures 6B–G; yellow circle) and unlesioned (Figures 6H–M) telencephalic hemispheres, respectively (One-way ANOVA; tukey's *post-hoc* tests for multiple comparisons; *p* < 0.05), but no change at any time post-injury in the optic tectum (Figures 6N–S) and cerebellum (Figures 6T–Y) compared to control (One-way ANOVA; tukey's *post-hoc* tests for multiple comparisons; *p* > 0.05). The difference in EdU volume between *Structure Analysis* and *Slice Analysis* likely reflects quantification of both the stem and immune cell response in the former, while during *Slice Analysis* we specifically targeting the neurogenic zones where mostly stem cells reside.

### Whole brain OPT scanning as a tool to understand stem cell niche development and for detecting changes in brain morphology

**Figure 7:**
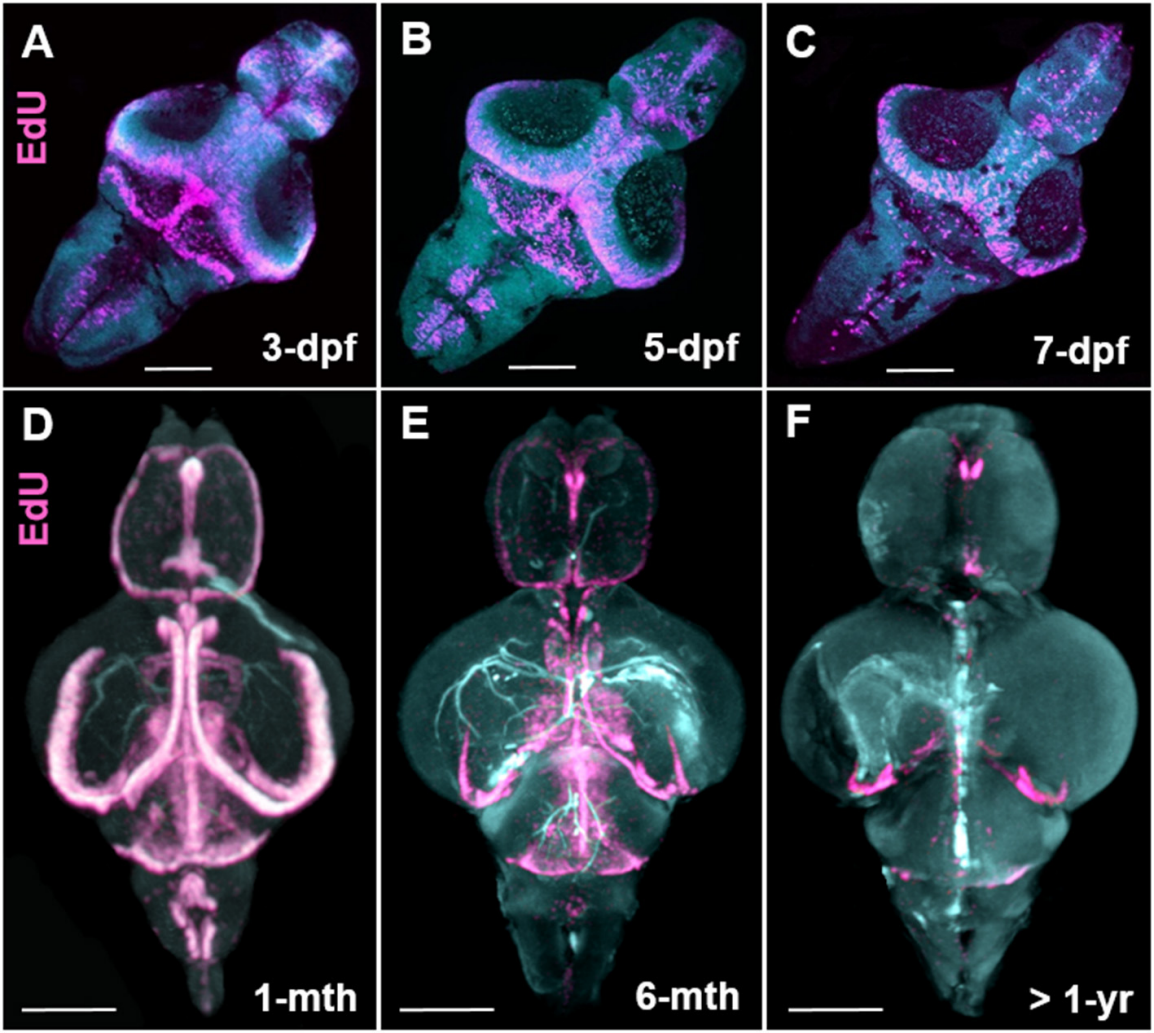
Stem cell niche development over zebrafish ontogeny. **(A–C)** Whole mount EdU (pink) staining and confocal imaging in transparent larvae at 3-, 5-, and 7-dpf visualized in 3-D using IMARIS, displaying the early pattern of cell proliferation throughout the developing zebrafish brain. **(D–F)** Whole mount EdU (pink) staining and OPT scanning in juvenile (**D**), adult (**E**), and senescent (**F**) brains visualized in 3-D using IMARIS, depicting a reduction in constitutive cell proliferation within stem cell niches situated along the A-P brain axis. Dpf, days post fertilization; mth, month; yr, year. Scale bars = 500 μm.

**Figure 8:**
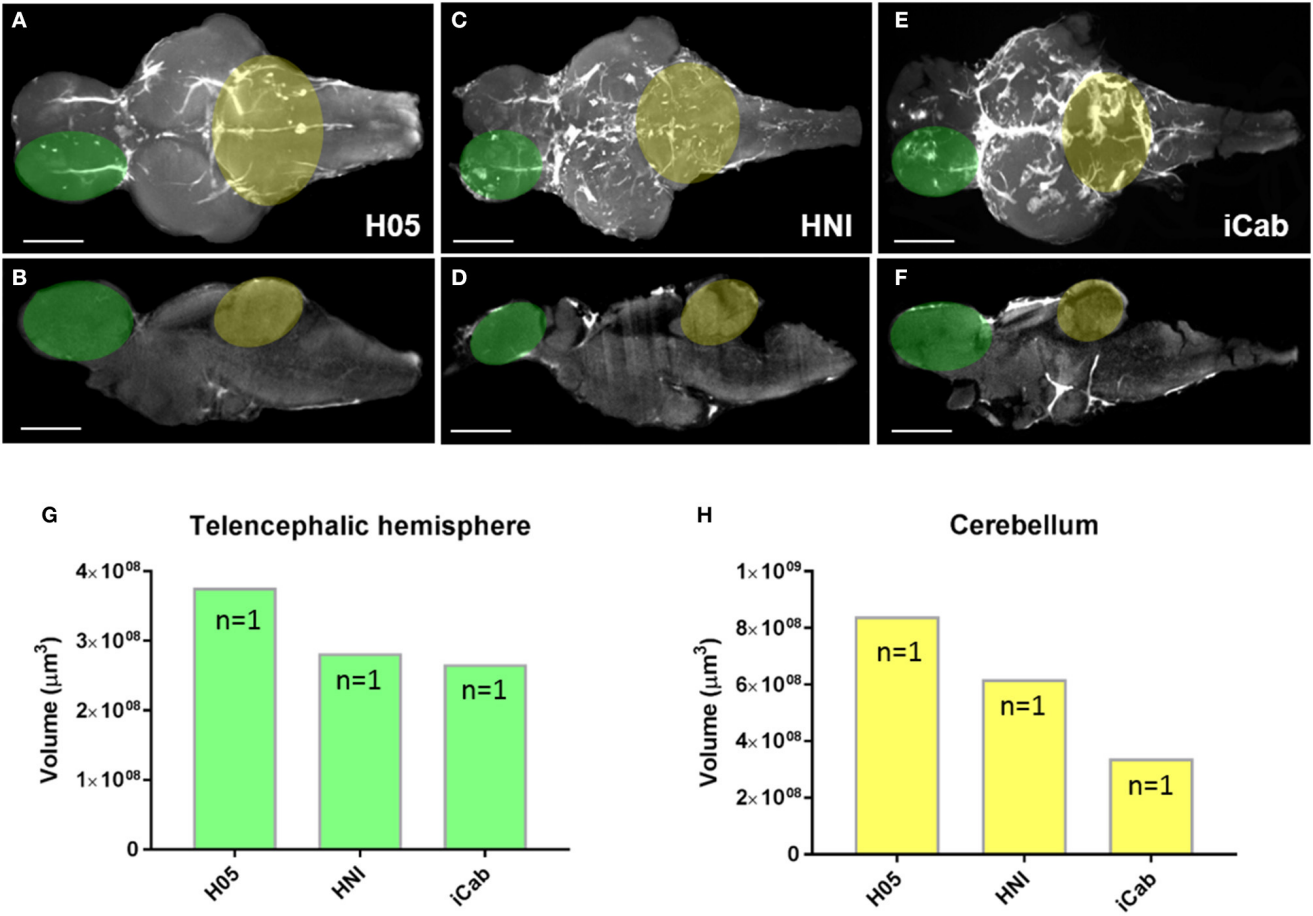
Morphological variation in brain structures across inbred medaka strains. **(A–F)** Adult medaka brains in dorsal and mid-sagittal views from the H05 **(A,B)**, HNI **(C,D)**, and iCab **(E,F)** inbred strains scanned using autofluorescence to investigate morphological variation in the growth/volume of major brain structures. The white region seen on brains depicts autofluorescence of vasculature (H05), or additional pigment left on the brain at the time of imaging (HNI, iCab). Green and yellow overlays on brains denote the neuroanatomical structures used for volume calculations shown in **(G,H)**. **(G,H)** Example volume calculation using IMARIS comparing the size of a single telencephalic hemisphere (**G**, green) and cerebellum (**H**, yellow) across the three inbred medaka strains. Scale bars = 500 μm.

How cell proliferation during early brain growth leads to the adult pattern of proliferative/neurogenic zones remains largely unexplored. Here we show that by taking advantage of the transparency of the larval zebrafish brain for whole brain confocal imaging of EdU (Figures 7A–C) and the application of our EdU OPT pipeline for the juvenile to senescent brain (Figures 7D–F) it is possible to seamlessly track how adult stem cell niches develop, how patterns of cell proliferation change, and assess when cells enter a quiescent state by combining additional markers (data not shown). This approach could be fruitful for comparative studies between other leading teleost models to uncover species-specific differences in stem cell niche development and aging. Additionally, performing the present OPT pipeline in teleost models, such as the medaka, whose brain is comparable in size, offers a novel approach to analyse changes in brain morphology across various inbred strains using autofluorescent scans only (Ishikawa et al., 1999; Spivakov et al., 2014). Results of our OPT scans of three different inbred strains of medaka, H05, HNI, and iCab (Figures 8A–F), show that differences in the volume of specific brain structures (Figures 8G,H) can be detected and quantified for downstream statistical analysis. Combining this morphological analysis with EdU labeling could further uncover how proliferative patterns are related to the development of smaller or larger brain structures, unveiling how genetic variation regulates brain growth over vertebrate ontogeny.

## Discussion

Three-dimensional fluorescent macro-imaging of whole organs is becoming commonplace in biology, providing new perspectives on how organs, tissues, and cells develop and respond to trauma or disease (Lupperger et al., 2017; Whitehead et al., 2017). Within the field of neuroscience, the existence of few practical methods to visualize the mature vertebrate brain in a 3-D context has hindered progress in understanding global patterns of change across cell populations following manipulation. The protocol illustrated here provides the neuroscience community with an innovative, simple method to visualize brain-wide patterns of cell proliferation along with brain morphology by taking advantage of the small size of the adult brain of the zebrafish model, the consistency of EdU labeling, and the isotropic nature of Optical Projection Tomography (OPT). Additionally, our OPT pipeline allows for the possibility of combining EdU staining with transgenic reporter lines and/or antibodies. Moreover, we demonstrate that reconstructed datasets can be easily quantified using freeware such as FIJI/IMAGEJ using EdU intensity or volume as output to examine broad patterns of change or within specific neuroanatomical domains of interest. The data can also be used as a building block to create a 3-D atlas and for standardization of expression patterns. Collectively, these features will establish this protocol as a valuable tool in small teleost models such as the zebrafish, to unveil new clues underlying brain-wide stem cell behavior during the regenerative process, new information underpinning systemic cell signaling of stem and immune cell populations, as well as tracking stem cell niche development and changes in brain morphology.

The successful labeling of proliferating stem/progenitor cells in the adult zebrafish brain in the current protocol depends largely on the properties of 5-ethynyl-2′-deoxyuridine (EdU). EdU has evolved as a modern alternative to the use of previous thymidine analogs, 5-bromo-2'-deoxyuridine (BrdU), 5-chloro-2'-deoxyuridine (CldU), and 5-iodo-2'-deoxyuridine (IdU), for labeling active DNA synthesis (*S*-phase) of the cell cycle (Salic and Mitchison, 2008). Moreover, unlike BrdU protocols, EdU labeling does not require DNA denaturation using harsh chemicals such as hydrochloric acid that often degrades tissue (Buck et al., 2008). Rather, EdU staining uses a rapid click-chemistry reaction between an azide (part of staining solution) and an alkyne (bound to EdU), allowing cryosectioned tissue to be labeled at room temperature in <30-min, and in the case of the whole adult zebrafish brain, within 4-days. The small size of the Alexa Fluor azide is readily accessible to the DNA, unlike larger anti-BrdU primary antibodies, making it well suited for penetration into thick tissue during the staining process. Importantly, EdU fluorescence is not degraded by methanol dehydration or BABB clearing like many other markers (Figures 2I–K), making it ideal for whole brain imaging and resulting in consistent labeling patterns in line with conventional cryosectioned tissue (Figures 2E,G).

Effective use of this protocol for sample preparation of zebrafish or other small vertebrates with comparable adult brain size requires attention and expertise at a number of different stages. Proper EdU administration is crucial for visualization of this marker during OPT imaging, and it is most cost effective to inject intraperitoneally as described. However, bath application in EdU can also be considered using the same chase periods presented here. In cases where this protocol is considered in juvenile animals, intraperitoneal microinjection of EdU or bath application must be performed due to the small size of fish. Proceeding EdU chase periods, brain dissection may be one of the more technically challenging steps. Nevertheless, time should be taken to remove the entire brain intact and ensure no debris (i.e., pigment, blood, tissue) remains on the surface before transfer into fixative (Figures 1C,D). Debris will impede the passage of light through the sample during OPT imaging, resulting in poor reconstructions and inaccurate analysis.

Without exception, embedding the EdU stained brains in the 6-well plates is the most important step of the protocol. Poor embedding will result in unusable samples even if the EdU labeling appears unspoiled. Thus, it is advisable that when starting this protocol for the first time, only 1–2 brain samples are embedding at a time in low melting agarose (Figure 1F). Agarose embedding has long been used in zebrafish research for histology of fixed specimens (ZFIN; Tsao-Wu et al., 1998; Copper et al., 2017) or live *in vivo* confocal imaging of larvae (Kaufmann et al., 2012), and is commonly used during sample preparation for OPT scanning across leading animals models, including but not limited to adult *Drosophila* (McGurk et al., 2007), mouse embryos and kidney (Sharpe et al., 2002; Short et al., 2010), and human brain tissue (Kerwin et al., 2004). Once the agarose begins to set there is a very narrow window of time to orient brains properly and smoothly without damage. Paramount is that brain samples do not fall to the bottom of the well, as this will place them outside the field of view when OPT imaging. Taking time to observe the samples in the z-plane of the well by looking at the side of the 6-well plate provides a good indication if brain samples are properly oriented. Lastly, before trimming the block always confirm which type of OPT mount will be utilized as this might require modifications to how the block is trimmed. Moreover, applying this protocol to other types of imaging, such as light-sheet microscopy, may necessitate slight deviations.

Those interested in taking advantage of the present protocol should make it common practice to run preliminary experiments and determine experimental parameters for downstream analysis prior to commencing OPT imaging. The importance of this cannot be overlooked, especially if the intensity of EdU is to be a measured output as we show with our *Histogram Analysis* (Figure 4). For instance, lesioned brains have considerably greater EdU intensity compared to control levels as a consequence of greater cell proliferation following injury (see Figures 4C, 6B–F). As a result, confirming a consistent level of exposure for both treatments that does not lose data in controls, nor over-expose data in the damaged brains must be done *a priori*. Similar to confocal imaging of immunohistochemical stained tissue, each sample will have slight variations in EdU intensity. Using a sample size of *n* = 5–10 animals in most cases allows for a reliable pattern to be extracted following post-processing.

Whole organ imaging produces large datasets (>1 GB) and often requires specialized quantification tools and powerful software to extract cell counts, staining patterns, or global trends from 3-D tissue. FIJI/IMAGEJ, IMARIS, and MATLAB are some of the more commonly available software programs that can be used to create 2-D or 3-D analysis methods to quantify reconstructed OPT datasets. In this paper we showcase three simple downstream analysis methods we developed exclusively in FIJI/IMAGEJ to examine the A-P profile of EdU intensity (*Histogram Analysis*: Figure 4) and EdU volume in large tissue regions of the adult brain (*Structure Analysis*: Figure 5) or tightly demarcated adult proliferation zones (*Slice Analysis*: Figure 6) following telencephalic lesion. While sophisticated computational methods to normalize 3-D expression data for standardized neuroanatomical maps are emerging in the larval zebrafish following live imaging (Randlett et al., 2015), not surprisingly, few analysis pipelines currently exist to analyze 3-D output derived from the adult zebrafish brain (Lupperger et al., 2017).

Although some modern OPT scanners are capable of single cell resolution, most provide datasets with only near cellular resolution. Undoubtedly this has limitations on how downstream analysis is performed on reconstructed OPT output. Given this, we view our EdU staining OPT pipeline as a starting point to visualize brain-wide trends in cell proliferation under varying conditions to identify neuroanatomical domains of interest that can subsequently be studied at the single cell level using immunohistochemistry, *in situ* hybridization, or electron microscopy on sectioned tissue. Analysis of mouse kidney has previously shown that OPT tissue is compatible with physical sectioning for immunostaining and transmission EM, allowing users to seamlessly move from one technique to the next within a single sample (Combes et al., 2014). Here we illustrate that both EdU intensity and EdU volume are informative and reliable methods to represent and quantify OPT data to investigate systemic changes in the CNS post-injury. In particular, we highlight that EdU volume derived from OPT scans is a statistically detectable readout to compare changes in EdU staining between the uninjured and injured brain.

Our combined *Histogram* and *Structure* analyses bring to light that the systemic response of cell proliferation to forebrain injury is apparent along the entire length of the neuro-axis and peaks at 1-dpl across all structures distal to the lesioned hemisphere (Figures 4C, 6B–I). However, these analyses encompass all cells that have entered a proliferative state, which is known to include a significant population of immune cells that are activated upon injury and present in the tissue parenchyma (Kroehne et al., 2011; Kyritsis et al., 2012; Kaslin et al., 2017). Comparing these findings with the results from our *Slice Analysis*, we observed that lesion-induced signals do not appear to modulate the degree of cell proliferation in the proliferative domains of posteriorly located tectal and cerebellar structures (Figures 6N–Y), but rather remains proximal to the lesioned and unlesioned hemisphere (Figures 6B–M). The dichotomy in the pattern of change in EdU observed between *Histogram/Structure Analysis* and *Slice Analysis* imply that cues from the lesion site differentially activate populations of adult neural stem cells in stem cell niches positioned along the A-P axis, but trigger global proliferation of leukocytes in the brain parenchyma.

Beyond studies of global changes in EdU following traumatic brain injury, we show that our OPT protocol has merit for uncovering new clues related to stem cell niche development and brain morphology. By combining larval whole brain confocal imaging of EdU (Figures 7A–C) with EdU labeling using our OPT pipeline (Figures 7D–F) we are now able to visualize the localization of cell proliferation over the full spectrum of zebrafish development into senescence to track the proliferative status of individual life-long proliferation/growth zones. We see this work as a fundamental starting point to transition towards being able to obtain isotropic 3-D data of specific gene expression patterns in the adult CNS that can be used to create standardized brain maps and bridge the gap between embryonic, larval, juvenile and adult shape and morphology. Likewise, coupling whole brain imaging of developing adult stem cell niches with cell-specific markers, methods such as immuno-correlative light and electron microscopy, and niche-specific transcriptomics will be a powerful approach to dissect how distinct niches are constructed and regulated at key developmental milestones. Secondly, we show that our OPT pipeline can readily be adapted to other small experimental fish models, such as the medaka, for comparative studies of brain morphology and cell proliferation (data not shown). Taking advantage of brain autofluorescence that can be imaged during OPT scans, we reveal that this is a tractable method to obtain morphometric scans of brain volume for quantitative analysis. By directly testing the feasibility of this approach in three inbred strains of medaka (Figures 8A–F), we demonstrate that differences in the volume of distinct brain structures can be quantified using IMARIS volumetric algorithms (Figures 8G,H). We envision this approach to be highly desirable to the medaka community to progress current knowledge on the genetic basis of brain development as a result of the high tolerance of this species to inbreeding (Kirchmaier et al., 2015).

Optical Projection Tomography was first designed to study gene expression patterns in the developing mouse embryo (Sharpe, 2003). Over the last 15-years OPT has played a pivotal role in answering a diversity of biological questions primarily in rodent models (Vinegoni et al., 2009; Short et al., 2010; Jeansson et al., 2011; Gleave et al., 2012, 2013; Anderson et al., 2013; Combes et al., 2014; Short and Smyth, 2016, 2017), using commercially available or custom built OPT systems (Wong et al., 2013). Here, we have optimized an EdU sample preparation pipeline for the adult zebrafish brain taking advantage of the tomographic imaging of OPT with the aim of expanding our knowledge of adult stem cell behavior following traumatic brain injury. We show that output from OPT scans can be examined quantitatively using FIJI/IMAGEJ to identify systemic changes in cell proliferation post-injury, and that our OPT pipeline would serve as a beneficial imaging tool to track stem cell niche development over ontogeny and study changes in brain morphology. Moving forward we welcome collaborations from members of the teleost community (zebrafish, medaka, or other) and hope this protocol will be of practical use for many laboratories.

## Author contributions

The protocol presented here was developed by BL and JK for zebrafish, in collaboration with FL for its application with medaka. BL was responsible for drafting the manuscript and figures with conceptual input from both JK and FL. AD was responsible for EdU staining and larval whole brain confocal imaging in zebrafish. AD and BL performed all revisions to figures and the inclusion of additional data in the manuscript. BL and JK were responsible for the creation of supplementary videos accompanying the manuscript.

### Conflict of interest statement

The authors declare that the research was conducted in the absence of any commercial or financial relationships that could be construed as a potential conflict of interest. The reviewer GRM and handling Editor declared their shared affiliation.

## Supplementary material

Supplementary Video 1 Instructional video explaining and demonstrating key steps in whole brain staining, embedding and clearing pipeline for adult zebrafish.

Supplementary Video 2 OPT scanned and reconstructed adult zebrafish brain following a 4-h EdU pulse displaying the stereotypical pattern of adult stem cell niche proliferation along the anterior-posterior neuro-axis.

Supplementary Video 3 3-D reconstructed and rendered adult zebrafish brain showing an overlay of brain volume OPT scanned using autofluorescence from the 488 channel (green) and EdU labeling using the 555 laser (red).
